# Complicated Cerebral Venous Thrombosis During the First Trimester of Pregnancy

**DOI:** 10.7759/cureus.10683

**Published:** 2020-09-27

**Authors:** Raphael Bertani, Renato B Rodrigues, Stefan W Koester, Fernando Augusto Vasconcelos, Ruy Monteiro

**Affiliations:** 1 Neurological Surgery, Hospital Municipal Miguel Couto, Rio de Janeiro, BRA; 2 Emergency Medicine, Estácio de Sá University Medical School, Rio de Janeiro, BRA; 3 Surgery, Federal Fluminense University, Niterói, BRA; 4 Medicine, Vanderbilt University Medical Center, Nashville, USA

**Keywords:** decompressive hemicraniectomy, craniectomy, first trimester pregnancy, cerebral venous thrombosis, thrombosis in pregnancy, cerebral venous sinus thrombosis (cvst), anticoagulation in pregnancy

## Abstract

Pregnancy and puerperium are known conditions associated with venous thrombotic events, which may present atypically in cases such as cerebral venous thrombosis. Since these are uncommon events, there is a paucity of reports and protocols for the management of these patients, resulting in no clear consensus in the literature. We report a case of a woman, nine weeks pregnant, who developed thrombosis of the right transverse and superior sagittal sinuses. Our diagnosis was made with computed tomography angiography, and due to a significant midline shift, an emergency decompressive hemicraniectomy was required. Although medical and surgical therapies for intracranial hypertension and anticoagulation were optimized in accordance with current medical literature, the patient suffered a spontaneous abortion and remained with significant neurological sequelae.

## Introduction

Cerebral venous thrombosis (CVT) is a rare condition that can lead to cerebral infarction through increasing venous and capillary pressure, therefore disrupting cerebral perfusion [1]. It is more prevalent in females and associated with prothrombotic risk factors such as surgery, trauma, sepsis, cancer, thrombophilia, anti-phospholipid syndrome, puerperium, and pregnancy [1]. The third trimester and the first month after childbirth are the periods with higher risks for pregnancy-related-CVT [2]. The disease arises from an imbalance between coagulation and anticoagulation systems which generates blockage of venous outflow and metabolic disturbance of cerebral cells. High capillary pressures favor the hemorrhagic transformation that occurs more frequently than in other ischemic conditions. Ischemic aggression is usually reversible as treatment with heparin (the first line of treatment) interrupt thrombotic progression and allows restoration of venous outflow by endogenous anticoagulation system [3]. Typically, these patients experience good outcomes as, nowadays, CVT treatment evolves with low mortality rates (8% to 14%) and very few individuals (8.7%) with permanent neurological deficits [3]. Nevertheless, some cases have poor outcomes and develop intracranial hypertension requiring emergency craniectomy in addition to clinical anticoagulation and intracranial hypertension treatments [4].

Additionally, pregnancy-related-CVT carries an additional concern regarding the health of the child. However, as it only occurs around 0.03% of all pregnancies, substantial clinical trials are absent and only a few reported cases on pregnancy can be found, resulting in a lack of guidance for these conditions [2]. This article plays an important role in discussing current controversies in the literature about contemporary therapy for CVT, especially on early pregnancy.

## Case presentation

A 28-year-old female patient, nine weeks pregnant, was admitted to the emergency department, brought by family members who reported nausea, emesis, confusion, and impaired balance. She scored 13 (M6 V3 E4) points on the Glasgow coma scale (GCS). According to family members, the onset of symptoms was 24 hours before her admission. Due to a persistent history of emesis throughout the pregnancy and an exacerbation of nausea and vomiting on the last 24 hours, Wernicke-Korsakoff syndrome secondary to Hyperemesis Gravidarum was suspected. The patient was treated with the restitution of B1 vitamin (thiamine) and intravenous 5% glucose solution. As the symptoms progressed and the patient deteriorated (GCS of 6), the neurosurgery team was requested for a consultation.

**Figure 1:**
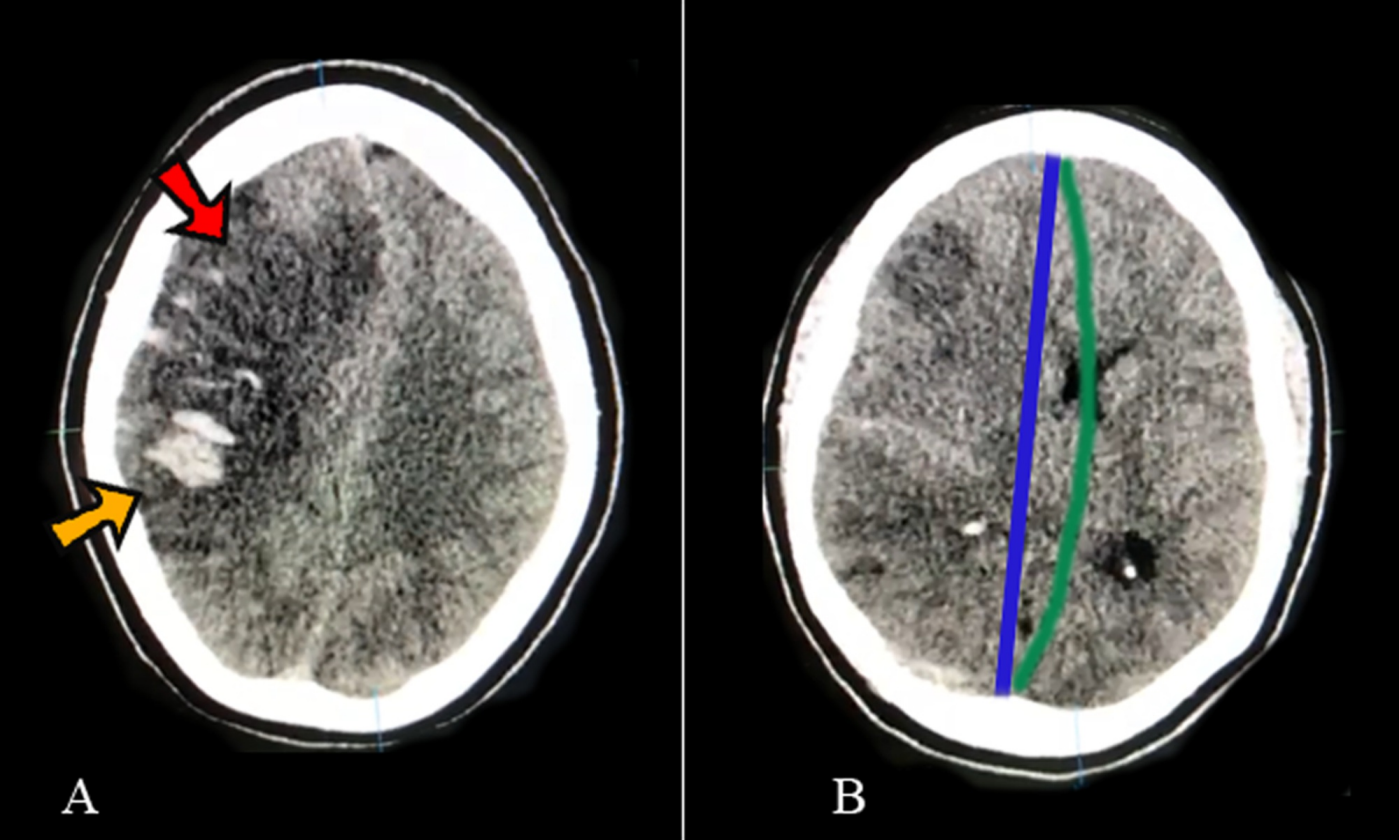
Pre-operative computerized tomography (CT) scans Pre-operative computerized tomography scans show on A: Large frontoparietal hypodensity (red arrow) with hyperdense areas (yellow arrow) suggestive of ischemic stroke with hemorrhagic transformation and on B: Midline shift measuring 8.5mm, suggestive of cerebral edema (>5mm). The midline is marked with a blue line and the midline shift with a green line. All of these can be indirect signs of cerebral venous thrombosis (CVT)

A computed tomography scan (CT) revealed extensive left parietotemporal hypodensity (approximately 15 x 7.6 x 7.7cm) with a hyperdense center, suggestive of ischemia with hemorrhagic transformation, edema, and a midline shift (8.5 mm) (Figure 1). The patient was stabilized and a decompressive hemicraniectomy was performed due to significant midline shift and rapid neurological deterioration.

**Figure 2:**
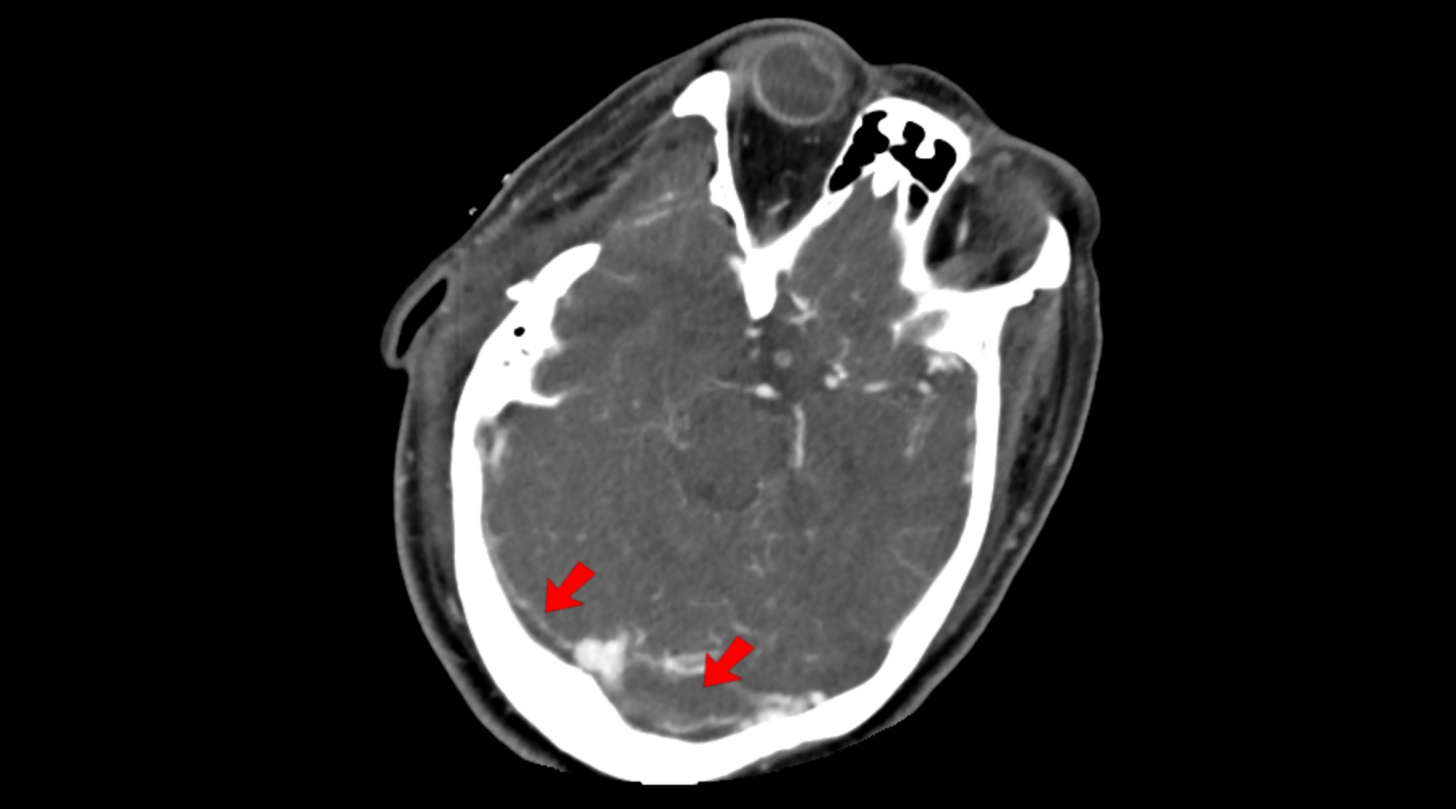
Post-operative computerized tomography angiogram Post-operative computerized tomography angiogram showing images resembling transverse sinus filling defects (red arrows), suggestive of cerebral venous sinus thrombosis

Post-operatively, standard medical therapy for intracranial hypertension (IC) was continued in the intensive care unit (ICU). A CT angiography was also requested, which revealed a significant filling defect on the transverse sinuses (Figure 2). Continuous unfractionated heparin was initiated in 48 hours with anticoagulant dosage (70U/Kg in bolus followed by a maintenance of 12U/kg per day for 14 days). The team chose unfractionated heparin over low-molecular-weight (LMW) heparin due to important hemorrhagic transformation on CT and, therefore, better reversibility with protamine in the case of hemorrhagic progression. She was also monitored with partial thromboplastin time measured every six hours with a target of 60 to 90 seconds. Post-operative CT scans did not demonstrate hemorrhagic progression.

Two days after arriving at the ICU, the patient experienced vaginal bleeding and was consulted by obstetrics-gynecology. Upon examination, blood clots were apparent in the vaginal canal, with dilation of the external orifice and no evidence of any infectious process overlying this event. The emergency ultrasonography showed ovular remains inside the uterus cavity compatible with spontaneous abortion and intravenous misoprostol was started. A blood transfusion of red cell concentrates was administered due to significant acute anemia (hemoglobin level of 6.5 g/dL). The patient restored hemoglobin levels higher than 7g/dL needing no further hemotherapy.

**Figure 3:**
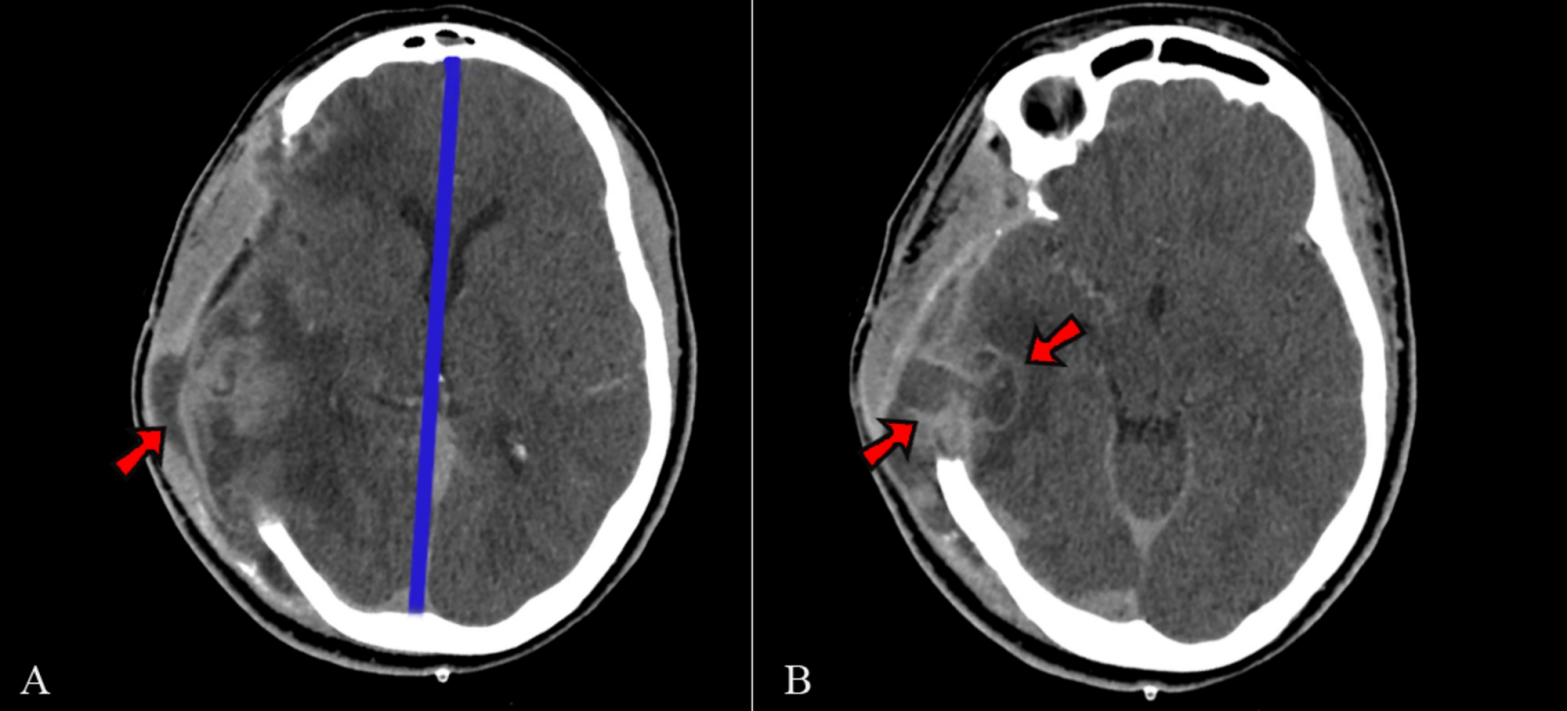
Post operative imaging showing development of surgical site infection A: Imaging shows purulent fluid (arrow). With improving of cerebral edema, the blue line shows improvement of midline shift and B: It can be seen that the fluid extends into the parenchyma, displaying an image suggestive of a parenchymal abscess (arrows), surrounded by a hypodense halo (edema)

After follow-up by the neurosurgery and obstetric teams, the patient progressed well with significant improvement (GCS 15). There was also interruption of vaginal bleeding, uterine colon opening, and normalization of the ultrasonography. With regards to neurological outcome within seven days, she showed right hemiplegia with a Medical Research Council Scale for Muscle Strength (MRC) grade of one for the left upper limb and three for the left bottom limb and significant speech deficit, being capable of verbalizing only short words. Fifteen days after the surgery and seven days after been sent to the wards, she started showing high fever (38.8 °C) with a worsening of general status. A purulent secretion was observed on the surgical site, as well as on imaging (Figure 3). The patient was submitted to surgical wound revision (after 48 hours of heparin suspension) for debridement of the surgical site. Antibiotics were started with intravenous meropenem (500mg every 8 hours) and amikacin (5mg/kg every 8 hours) according to the hospital microbiological profile.

**Figure 4:**
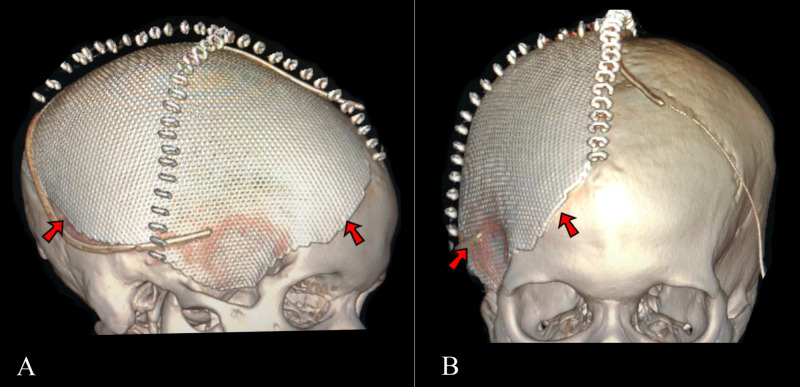
3D Reconstruction of post-cranioplasty computerized tomography scans 3D reconstruction showing cranioplasty with a titanium mesh prosthetic (red arrows), on a lateral view (A) and frontal view (B)

Antibiotics were maintained for 21 days, respecting bacterial cultures and antimicrobial sensitivity with clinical improvement and symptom regression. Following surgical wound debridement, the team introduced oral rivaroxaban (20 mg daily) and the patient was discharged maintaining hemiplegia and aphasia after antibiotics conclusion. Rivaroxaban was maintained for six months and was suspended 72 hours before cranioplasty with no further complications (Figure 4). At this time, the patient still presented residual motor deficits with substantial improvement of strength (MRC 3) on the lower left limb. One year following the initial presentation, she remained with strength scores of 3 on the left lower limb and 1 on the left upper limb. Her Glasgow outcome extended score (GOSE) was 6.

## Discussion

Although CVT is a rare condition [1], it should be considered as a differential diagnosis especially when a pregnant woman presents with an acute neurological deficit that raises suspicion for ischemic disease.

As previously mentioned, the disease arises from an imbalance of the anticoagulation and coagulation systems with obstruction of venous outflow [3]. The principal sites for flow obstructions seem to be the junctions of cerebral veins and the major cerebral sinuses with a predilection for the superior sagittal (SSS) and transverse sinuses [5,6]. 

Even though it can progress with large areas of ischemia and hemorrhagic progression, in general, metabolic disturbance of cells is reversible and, with adequate treatment, patients have a relatively good prognosis, even when requiring a decompressive craniectomy [4]. Other factors that can predict good prognostic outcomes are the absence of hemorrhagic transformation and early reestablishment of venous outflow [5, 7]. When considering higher postoperative mortality, other factors have been identified as risk factors, such as age older than 40 years, the involvement of deep venous systems, more than two dural sinuses involved, and delay of treatment for more than 12 hours [6].

Clinical presentation can be obscure and often confused with other common pregnancy complaints. Headache, lethargy, vomiting, nausea, aphasia, motor deficit, and seizures are common manifestations [3]. These patients progress quickly (if not present since admission) to a deteriorated neurological state with signs of cranial hypertension and/or herniation, such as anisocoria [5, 6]. Radiological signs can be divided into two groups: indirect signs (edema, ischemic hypodensity and hemorrhage), and direct signs (signs of the empty delta and of the cord). The cord sign is an early sign (usually found on the first two weeks) caused by hyperdensity of the sinuses and cerebral veins (deep or cortical veins). The empty delta sign is mostly a delayed sign (usually found after two weeks of onset) of a triangular hypodensity, not enhanced by contrast, and commonly at the SSS [8].

Anticoagulation with heparin constitutes the first line of treatment since it stops the progression of thrombosis and allows the endogenous anticoagulation system to restore vascular patency. One of these treatments, unfractionated heparin, is a drug accompanied with partial thromboplastin time (PTTa) and our team chose to administer this treatment due to its reversibility with protamine in the event of a hemorrhagic transformation. 

Thrombolytic drugs, such as streptokinase or tissue plasminogen activators, seem to be an acceptable choice, considering the risks of serious thrombotic events during pregnancy [9]. However, there is no clear evidence of any benefit of associating medical treatment with endovascular approaches [10]. Despite a low level of evidence, mechanical thrombectomy alone can also be considered for non-pregnant patients without immediate life-threatening risks [11].

Decompressive craniectomy has shown to be beneficial to critically-ill patients with CVT presenting radiological and clinical features of mass effect with signs of brainstem dysfunction and refractory intracranial hypertension [5]. Time to begin or return anticoagulation is one of the main controversial points when a decompressive craniectomy is required for treatment of CVT. Current medical literature has reached a consensus about safety of returning or starting heparin after 24 to 48 hours after surgery [7, 12]. 

Circumstances such as pregnancy require special attention and consideration of differential treatment options. There is no proven evidence of benefit in abortion regarding the mother’s or child’s health over not interrupting pregnancy [2,6]. However, we do recommend a multi-specialty approach where an obstetrics team can decide what is best for the mother’s health. In some previous cases, where the fetus was sufficiently mature, a cesarean was performed as a way of preserving clinical stability (older than 32 weeks) [2,12]. Our team highlights that in cases other than an elective anticipated delivery, appropriate prophylaxis should be performed, including: 1) Betamethasone, two doses of 12mg, apart 24 hours, on 24-34 weeks pregnancies and 2) Magnesium sulfate on pregnancies earlier than 33 to 31 weeks [13, 14]. 

Regarding early pregnancy, a case report described how a 22-year-old woman, pregnant of 12 weeks, developed occlusion of the right transverse sinus causing cerebral infarction with hemorrhagic transformation and edema of the right temporal lobe [2]. On that occasion, pregnancy continued normally with the patient giving birth after six months post-presentation. We believe that the fact that our patient progressed to a critically ill state may have had a deleterious effect on fetal health.

As reported extensively in the literature, other prothrombotic conditions can be overlapped with pregnancy and puerperium [9]. When seeing such patients, obtaining a scrupulous history of past clinical conditions, medications used, symptoms of rheumatologic diseases, previous thrombotic events, and family history of thrombotic events can be valuable. In table 1 we present the most prevalent conditions related to CVT and methods for diagnosis that should be individualized by clinical suspicions. Further pregnancies are not contraindicated and when avoidance of oral contraceptives and correct prophylaxis with LMWH occurs, the risk of subsequent recurrence is considerably reduced [2,12].

**Table 1:** Conditions may increase risk of thrombotic events A summary of possible conditions that may increase the risk of thrombotic events with their clinical manifestations, diagnosis, and management. *CVT: cerebral venous thrombosis; ECL: erythematous cutaneous lupus; APS: antiphospholipid syndrome; ANF: antinuclear factor; ASA: acetylsalicylic acid; LMWH: low-molecular-weight heparin; INR: international normalized ratio; PTTa: partial thromboplastin time.

Condition		Clinical Manifestations		Diagnostic	Management
Pregnancy		Menstrual period delay, mastalgia, nausea and vomiting, odor aversion, and others		Beta-HcG tests	Further pregnancies are not contraindicated in case of CVT. There is no indication of interrupting pregnancy during CVT, except on mother life-threatening risks
Use of Oral Contraceptives		-		Clinical History	If there were previous episodes of CVT, use should be contraindicated
ECL and APS		Malar rash, alopecia, oral ulcers, proteinuria, arthritis, anemia, and others for ECL. History of previous thromboembolic events and abortion for APS		ANF ≥ 1:80 + Clinical and laboratory Criteria (EULAR/ACR 2019). Vascular Thrombosis or Gestational morbidity with antiphospholipid antibodies demonstration	Treat ECL following medical recommendations. In case of any previous thrombotic events, such as CVT, and confirmed diagnosis of APS, patients should receive prophylaxis on next pregnancy with ASA and LMWH
Thrombophilias		Thrombosis on patients younger than 45 years; recurrence; family history of thrombosis; migrainous thrombotic events		Platelet counts, INR, PTTa. Consider investigate most prevalent thrombophilias: factor V Leiden, mutation on protein C, S and prothrombin.	Chronic treatment for recurrence of thrombosis with anticoagulants such as Vitamin K antagonist (except on pregnancy)
Infection and Sepsis		Fever, tachycardia, hypotension, tachypnea, leukocytosis, neutrophilia, leukopenia, lymphocytosis		Clinical examination. Require imaging and laboratory exams according to system signals and sypmtons (Chest radiography, urynalisis, microbiological cultures and others	Microbiological specific treatment. Suitable clinical care in case of sepsis or severe infection

Lastly, regarding the neurological deficits, there are no studies reporting long-term outcomes to our knowledge. Some studies have shown most patients have good outcomes (considered as Modified Ranking Scale grade of one or two) in the short-term (6months to one year) [2,6,12]. Further studies are necessary to identify patients with higher risks for poor outcomes.

## Conclusions

CVT is a rare cause of neurological deficit that must be considered when investigating pregnant patients. Considering CVT’s good prognosis when promptly treated, it is important to consider it as a differential diagnosis for neurological symptoms in this population and focus on an early approach. This may require a team effort between neurosurgeons, neurologists, obstetricians, and emergency clinicians to rapidly identify the above-mentioned symptoms and raise diagnostic suspicion for CVT, especially when investigation and treatment of other more prevalent conditions are not fruitful. Anticoagulation with LMWH is currently the first line of treatment, and surgical intervention can be performed in cases of mass effect with significant midline shift and neurological deterioration. Surgical intervention must be tailored to each case, with hematoma evacuation or either primary (at the time of hematoma evacuation) or secondary (in the setting of refractory intracranial hypertension) decompressive craniectomies being possible treatments. Although pregnancy can be maintained, the mother’s health should be the priority in case of complications. Larger studies are needed to build significant scientific evidence regarding outcomes and best practices regarding therapy for these cases.
